# A novel model of clinical reasoning: cognitive zipper model

**DOI:** 10.30476/jamp.2020.82230.1050

**Published:** 2020-04

**Authors:** SHAHRAM YAZDANI, MARYAM HOSEINI ABARDEH

**Affiliations:** 1 School of Medical Education, Shahid Beheshti University of Medical Sciences, Tehran, Iran

**Keywords:** Clinical decision making, Problem solving, Judgment

## Abstract

**Introduction:**

Clinical reasoning is a vital aspect of physician competence. It has been the subject of academic research for decades, and various models of clinical reasoning have been proposed. The aim of the present study was to develop a theoretical model of clinical reasoning.

**Methods:**

To conduct our study, we applied the process of theory synthesis in accordance with the Walker and Avant’s approach. First, we considered clinical reasoning as a focal concept of our study. Second, a search was carried out for the period 1984–2018, using the PubMed, Google Scholar, PsycINFO, ERIC, ScienceDirect and Web of Science databases to review the literature to identify factors related to the clinical reasoning and the nature of their relationships. Third, we organized clinical reasoning into an integrated and efficient representation of the clinical reasoning.

**Results:**

According to this study clinical reasoning is the iterative process of intermediation between the recalled clinical knowledge and the patient’s represented problem in the clinicians’ active memory. We analogize the process of clinical reasoning to the process of closure of a cognitive zipper. The recalled knowledge in clinician’s memory resembles to one side of zippers teeth and the evolving representation of the patient’s problem resembles the other side of zippers teeth. So, the results of this study are presented in three models: [1] multi-layer knowledge structure model, [2] problem representation model and [3] cognitive zipper model of diagnostic reasoning.

**Conclusion:**

We propose a developmental model of clinical reasoning. Several studies have tried to present models and theories to clarify clinical reasoning, but it seems that these theories and models could only explain part of this complex process and not the whole process. Cognitive zipper model, due to its developmental structure, can illustrate the clinical reasoning process in more details than other models do.

## Introduction

Clinical reasoning (CR) is an important part of physician competence ( 1 , 2 ), and has an important role in the physicians’ ability to make diagnoses and decisions ( 3 , 4 ). CR is a challenging, promising ( 5 ), and a complex ( 6 ), multidimensional ( 7 ), mostly invisible and poorly understood ( 8 ) process ( 9 ). It has been the subject of academic research for decades ( 10 , 11 ), and researchers have proposed various models of clinical reasoning. In recent years, for example, Haring and her colleagues have proposed a conceptual model for expert judgment of clinical reasoning by medical students. This model contains a set of indicators for clinical reasoning, which can be used to assess clinical reasoning in medical students ( 12 ). Jarodzka and Boshuizen have combined the model of Lesgold et al. with cognitive expertise models of Boshuizen &amp; Schmidt into one model in medicine. This model illustrats the process of clinical reasoning and includes gathering patient data for the diagnosis and intervention ( 13 ). Another popular model of clinical reasoning is dual process ( 14 ). Although, there are many various models of clinical reasoning in medical education literature, but no single theoretical model is enough for such a complex process as clinical reasoning. It is clear that constructing a model which encompasses all the richness and complexity of the process of clinical reasoning is difficult, but this model can be helpful in teaching, evaluating, and learning clinical reasoning ( 15 ). The aim of the present study was to develop a theoretical model of clinical reasoning, which can unify all the models, and theories of clinical reasoning.

## Methods

As Walker and Avant noted, the aim of theory synthesis is "to put disparate, but related, scientific information into a more theoretically organized form". We applied the three steps of theory synthesis with the approach of Walker and Avant, 1) "Specified the focal concept", 2) "Identify related factors and relationships", 3) "Construct an Integrated Representation" ( 16 ). 

**Step 1: Specified the focal concept**

Clinical reasoning was the researchers’ field of interest, and was specified as the focal concept. We moved out from the clinical reasoning to other related concepts. 

**Step 2: Identify related factors and relationships**

Walker and Avant described "focal concept guided a careful search and review of the literature, and the variables related to the focal concept and their relationships were identified during the review" ( 16 ). We conducted a search based on our keywords "clinical reasoning, diagnostic reasoning, therapeutic reasoning, clinical decision making, problem solving, theory, and model" in PubMed, Google Scholar, PsycINFO, ERIC, ScienceDirect and Web of Science databases. We found an extensive literature (n=280) from 1984 to 2018. After removing duplicated studies, the articles with a title and abstract (n=140) were reviewed by the researchers. The studies eligible to this review were those which presented a model or a theory of clinical reasoning, or a description of clinical reasoning and the related variables (n=47). All parts of articles were read by two authors to find relationship statements. The inclusion criteria of selecting the studies were: 1) published articles in English and Persian, and 2) published articles in the field of medicine. If the studies provided clinical reasoning models or theories in other fields (like nursing and optometry), examined the clinical reasoning in the field of artificial intelligence (like clinical decision support systems), and/or examined brain biology and brain functions (like functional magnetic resonance imaging studies), they were excluded.

**Step 3: Construct an Integrated Representation**

In this step, researchers decided how to depict their model, and the way the model is represented depends on the theorist's creativity ( 16 ). We used the Blalock's “mechanism of theoretical modeling", and organized variables that were more proximally related into a "block" and specified their interrelationships. In order to present the final model, we first described a summary of the variables which were identified during the review, and then presented a model to promote better understanding of the variable, and finally, the comprehensive model of clinical reasoning was presented.

## Results

After reviewing the literature (step two), two variables were found: knowledge structure and problem representation. These two variables encompassed a range of sub-variables which are illustrated in table 1, and described below in more details.

**Table 1:** Clinical reasoning variables and sub variables

Variables	Sub variables
Knowledge structures	Clinical knowledge
	Basic science
	Organizational knowledge
	Legal knowledge
	Socioeconomic knowledge Codified knowledge
	Tacit knowledge
Problem representation	Clinical findings
	Para clinical findings
	Health risk factors
	Socio economics factors
	Discriminants findings
	Non discriminant findings
	Concomitant findings
	Accidental findings

Researchers have shown that reasoning is highly dependent on a person's knowledge base ( 17 ), and the manner in which medical knowledge is structured in the minds of physicians and students is important for the quality of medical diagnosis ( 18 ). A lot of representational models have been suggested to explain the structuring of knowledge in cognitive psychology ( 19 ). Moreover, many theoretical frameworks have been developed to explain how knowledge is organized and applied in reasoning and problem solving ( 20 ). For example, Gruppen and Frohna presented mental structures that include categories, prototypes, instances, schemas, scripts, and networks ( 21 ). Feltovich and Barrows proposed the illness script as the knowledge structure that links clinically relevant information about general disease categories, specific examples of diseases, and conditions that enable diseases to grow in human beings ( 22 - 24 ). Schmidt and his colleagues proposed "a stage theory of clinical reasoning" ( 25 ). Harasym suggested that the knowledge structure change during the acquisition of expertise and proposed five knowledge structures, including reduced, dispersed, elaborated, scheme, and script structures ( 26 ). 

Medical knowledge is placed in two categories: clinical and basic science knowledge. Clinical knowledge includes the knowledge of the processes, and the findings associated with the disease. The knowledge of basic science comprises a mix of topics such as biochemistry, anatomy, and physiology ( 27 ). The biomedical science and clinical knowledge can be integrated into coherent knowledge structures that can support all levels of physician performance and patient management ( 28 , 29 ). Tacit and codified knowledge are two forms of knowledge, tacit knowledge being obtained through experience, and its verbalization and transfer are difficult ( 5 , 28 , 30 , 31 ). In contrast, codified knowledge can be officially written or presented. It is explicit, formal, and systematic knowledge that can be expressed by words, numbers, and scientific methods or universal principles, and can be easily transferred, stored, and remembered ( 30 ). We believe that medical knowledge can be put in five subject categories, including: clinical, basic science, organizational, legal, and socioeconomic knowledge (Table 2).

**Table 2:** The relationship of five subject categories of knowledge with two forms of knowledge

Subject categories of knowledge	Forms of knowledge	
	Tacit knowledge	Codified knowledge
Clinical knowledge	*	*
Basic science		*
Organizational knowledge	*	
Legal knowledge	*	
Socioeconomic knowledge	*	

### *The multi-layers model of knowledge structure*

In the proposed model, knowledge structures consist of six stages and three layers. These stages include reduced knowledge, dispersed knowledge, casual knowledge, elaborated causal knowledge, conceptual knowledge, and deep conceptual knowledge. The three layers consist of the baseline knowledge layer, the schema layer, and the script layer.

Throughout the process of expertise, new knowledge is dynamically added to prior knowledge, and the composition and arrangement of the baseline knowledge layer continuously change in a process that is known as weak restructuring. In this type of restructuring, sequential systems of concepts are different in terms of the relations that exist between the concepts ( 32 ), but the main concepts remain the same across the systems ( 33 ). The baseline knowledge layer evolves in six stages, during which the amount and types of knowledge units undergo certain changes. 

In the last two stages of this model, conceptual change and development of superordinate concepts (strong or radical restructuring of knowledge) result in two additional layers; these are correspondingly named schema and script.

**Stage 1- Reduced knowledge:** In this stage codified basic and clinical knowledge is limited, and mostly irrelevant, and therefore, inadequate to solve clinical problems.

**Stage 2- Dispersed knowledge:** In this stage, increased codified clinical knowledge about diseases and their clinical manifestations are usually adequate for solving clinical problems, but the process of reasoning is inefficient because of a high level of noise (irrelevant knowledge) and a poor structure of knowledge. Clinical knowledge and the knowledge of basic science are not linked with each other; they are dispersed. 

**Stage 3- Casual knowledge:** In this stage there is adequate codified clinical knowledge to solve clinical problems. This knowledge is loosely integrated with causal relationships (linear/chain integration), but the process of reasoning is still meddled with a high volume of irrelevant knowledge. In this stage, knowledge structure is fortified by adding some clinical tacit knowledge which is acquired through repeated exposure to patients. The relationship between basic science and clinical knowledge has been created as a result of the experience that has been gained by a clinician. Clinical experience results in primordial relations between tacit and codified knowledge, which is indicative of weak restructuring of knowledge.

**Stage 4- Elaborated casual knowledge:** In this stage the clinician possesses a lot of relevant codified and tacit knowledge about diseases and manifestations, and their irrelevant codified knowledge has been reduced. In addition to clinical tacit knowledge, other forms of tacit knowledge such as organizational and social tacit knowledge are acquired through experience in authentic clinical settings. The clinician’s causal, pathophysiological, and mechanism knowledge creates an intricate networking of baseline knowledge with many categorical relations. Although the development of the baseline knowledge will continue for another two stages (stage 5 and stage 6), the main structure of baseline knowledge layer is formed up to this stage. As a result of weak restructuring, the relation between different knowledge units becomes richer and more sophisticated in the baseline knowledge layer.

**Stage 5- Conceptual knowledge:** Conceptual knowledge is defined as "knowledge that is rich in relationships, and knowledge of concepts, including principles and definitions"(34). Throughout the path of study and practice, conceptual knowledge is affected by many instances of conceptual restructuring. Moreover, the development of conceptual knowledge includes a shift from the novice's detached and flat knowledge to the expert's multi-layers structures ( 28 ). We believe that strong/ radical restructuring is a dominant feature of this stage. Strong restructuring consists of changing individual concepts, explanatory mechanisms, and theory ( 32 ). In this type of restructuring, concepts change and may differ, they may be intertwined, and can emerge or disappear; the relations between these concepts have changed substantially ( 33 ). During this stage, the schema layer evolves as new layer of knowledge in addition to baseline knowledge layer (schema's baseline knowledge layer at this stage).

Layer 1- Schema's baseline knowledge layer: As a result of pruning of irrelevant knowledge, the increase in tacit knowledge (specially the acquisition of legal tacit knowledge) and continuing weak restructuring, the schema's baseline knowledge layer is more concise, relevant and operational.

Layer 2- Schema: In our view, schema is the knowledge structure that occurs sequentially, has an algorithm form, and is stored in the long-term memory. Its sequences include an assumption that is formed in mind or is something that a person thinks about, such as the inquiries and searches they have performed, the findings that they found during this inquiry, and the decisions that they have ultimately taken. The schema layer can be mounted on top of the baseline knowledge layer according to strong or radical restructuring and creates a new concept that called schema.

**Stage 6- Deep conceptual knowledge:** During this stage a new and different concept called script is created based on strong restricting of knowledge. In this stage knowledge is organized in three layers:

1. Script's baseline layer knowledge
2. Elaborated schema layer
3. Script layer

Layer 1- Script's baseline layer: In this stage, some changes occur in baseline knowledge layer according to weak restructuring. These changes lead to the formation of highly relevant, contextualized, and networked knowledge that has little irrelevant codified knowledge, and more tacit knowledge, especially economic tacit knowledge compared with the schema's baseline layer.

Layer 2- Elaborated schema layer: At this stage considerable amount of tacit knowledge is acquired through a wide range of experiences and is added to script's baseline layer, this tacit knowledge being organized and transformed into more elaborated and contextualized schemas in accordance with the process of strong restructuring ( 20 ). 

Layer 3- Script layer: Scripts are list-like knowledge structures. They are results of schemas repeatedly used for common situations ( 34 , 35 ). Each Script contains a package or a list of expectations regarding what people consider or pursue in certain situations. The script layer is mounted on the elaborated schema layer in accordance with the strong restructuring of dominantly tacit expert knowledge (Table 3).

**Table 3:** Weak restructuring

1	Decrease in irrelevant codified knowledge through selective forgetting of irrelevant knowledge	Pruning of Knowledge
2	Increase in relevant codified, focused and domain-specific knowledge through search of foreground knowledge	Grafting of Knowledge
3	Increase in relevant tacit knowledge through experiential learning	Environmental Adaptation of Knowledge
4	Reorganization of knowledge into meaningful chunks through repeated simultaneous recall of separate parts of knowledge.	Topiary of Knowledge

***Variable 2: Problem representation***

The important starting point of the clinical reasoning process is problem representation ( 20 ). Constructing a problem representation is a part of the problem solving process ( 36 ), and is "the key to problem solving" ( 37 ). A problem solver constructs a problem representation based on their domain specific knowledge ( 38 ). It is a temporary cognitive structure that has been formed from the integration of prior knowledge with situational and patient-derived information ( 20 , 23 , 39 ). As a result of organizing the patient’s information into meaningful structures in the memory, a problem representation is developed ( 38 ), and it changes upon the discovery of additional data ( 15 ). Problem representation is made at the beginning of the contact with the patient ( 20 ) as a one-sentence summary that defines the specific case in abstract terms and displays the translation of patient-specific details into abstract terms ( 40 ). It is made in working memory ( 41 ), and has dynamic nature ( 15 ), and plays an important role in problem solving ( 42 , 43 ). To form an integrated problem representation, physicians elicit the given and goal information and links it to their existing knowledge ( 44 ). They would have to elicit findings to generate a coherent problem representation. Findings form the elements of the problem representation and are the first medically meaningful units derived from observations ( 45 , 46 ). We can describe all known diseases in terms of about 5,000 to 7,000 distinct findings ( 46 ). Evans and Gadd presented a hierarchical structure for medical knowledge; they have suggested that there be a possibility of distinguishing between different levels of classification of medical knowledge which serves to solve the diagnostic problem. They labeled these levels as Empirium, Observation, Finding, Facet, Diagnosis, and Global Complex ( 47 , 48 ). We believe that what Evans and Gadd have called the hierarchical structure of medical knowledge is actually a model for representing the problem. We were inspired by Evans and Gadd’s model and have designed a problem representation model that will be presented in the next sections. We have defined types of the findings that form a problem representation and then described the model of problem representation. We have categorized the findings based on their content and their relation to the disease.

The findings may be related or unrelated to the disease. The content of findings may include clinical findings, Para-clinical findings, health risk factors ( 42 ) and socioeconomic factors. 

Findings unrelated to disease are divided into two subgroups: accidental and concomitant findings. These findings are the noises that disturb the correct diagnosis. Accidental findings are not clinically meaningful; for example, clinically unimportant shadows in the patient's chest X-Ray. Concomitant findings are the ones that may be clinically important but are not related to the current patient’s problem; for example, knee osteoarthritis in a patient that has referred to a physician for inguinal hernia. 

Findings related to disease help the physician identify and diagnose the illness. These findings are divided into two groups of discriminant and non-discriminant findings. Non-discriminant findings are part of the clinical picture of the patient’s current disease, but are not specific to the disease, such as mild fever in a patient with acute myocardial infarction. Discriminant findings are highly predictive and sometimes specific (pathognomonic) to the disease and differentiate the disease from other diseases, such as neck stiffness, which is a specific finding in meningitis.

### *Problem representation Model*

When a physician encounters a patient, depending on the level of his/her expertise, different kinds of representations of the patient's problem are formed in his/her mind. These steps are as follows.

**Stage1-Inadequate Sensory Representation:** This kind of representation is formed by the novice who describes the sensory data with minimal medical interpretation and sometimes uses the words of the patient himself for these descriptions. For example, a medical student may describe the "caput medusa" sign as "observable veins around umbilicus". In fact, the person only describes what he/she has seen.

**Stage 2- Symbolic Representation:** Sensory data are symbolically represented by theoretical medical knowledge and medical terminologies of signs, symptoms, and other findings. The patient's problem is represented as a list of manifestations; the physician sees the findings and names them with medical terms. 

**Stage 3- Extended Manifestation Representation:** The problem is expressed in a more or less related list of clinical findings. The physician considers a set of findings as the manifestation of the disease, and so for each finding, a list of differential diagnoses comes to his/her mind. 

**Stage 4- Focused Manifestation Representation:** The problem is expressed by an abridged list of clinically relevant findings. As the physician focuses on more relevant findings the number of differential diagnoses will decrease in his/her mind. 

**Stage5-Extended Diseases Representation:** The problem is represented as lists of possible diagnoses (or clinical categories). 

**Stage 6- Focused Disease Representation:** The patient’s clinical problem and surrounding conditions are represented into a single diagnosis. In this representation, in addition to clinical and Para-clinical findings, health risk factors and socioeconomic factors that differentiate the disease are also identified by the physician.

**Figure 1:**
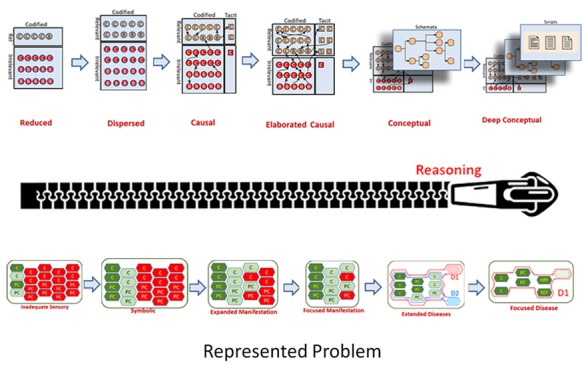
Cognitive zipper model

The knowledge structures and problem representations are two sides of a zipper, and the middle part of the zipper represents the mode of inquiry during the process of diagnostic reasoning. Given the interaction between knowledge structures and problem representations in the process of diagnostic reasoning, we believe that pre-existing knowledge structures and physician's perception of a patient's problem form as a problem representation in the physician’s mind when they confront a patient, and these are two main parts of the clinical reasoning process. As the result of interactions between knowledge structures and problem representations, the hypothesis is generated. There are two kinds of hypotheses, a posteriori hypothesis and a priori hypothesis. A posteriori hypothesis is generated after an observable phenomenon occurs. A priori hypothesis is a hypothesis generated prior to an inquiry process taking place. To illustrate the relationship between knowledge structures and the representation of the problem and how to formulate the process of clinical reasoning, we recommend a cognitive zipper model, as follows (Figure 1).

Depending on which level of expertise the person is at, the amount of closure and the openness of the zipper will change.

**Figure 2:**
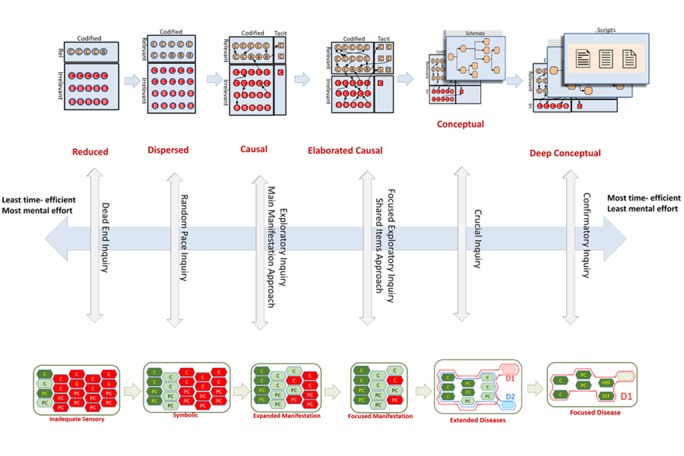
Modes of inquiry

There are six modes of inquiries in our model. These modes include dead end, random pace, exploratory, focused exploratory, crucial and confirmatory inquiry (Figure 2).

### *Dead End Inquiry*

In this mode of reasoning, clinical inquiry and representation of the patient’s problem are inadequate and because of the physician’s defective knowledge, no meaningful hypothesis is generated, and thus the physician’s cognitive endeavor leads to no diagnosis.

In this case, the lack of clinical skills results in incomplete history taking and physical exam and as Brenner showed in pre-algebra students, before the students build a problem representation in minds, they try to solve problems ( 49 ). We believe that problem representation in this mode of inquiry is inadequate and irrelevant narration of manifestations (inadequate sensory representation).

In this stage, the student’s knowledge about the clinical problem and its underlying disease is usually defective and unorganized (reduced knowledge structure). These two factors (inadequate sensory representation and reduced knowledge) lead to the production of wrong diagnostic hypotheses and the student struggles with these wrong diagnostic hypotheses through haphazard iterations of patient inquiry and repeatedly generates wrong diagnostic hypotheses. Usually, this mode of inquiry does not result in correct diagnosis, so we call this kind of inquiry as “dead end inquiry”.

### *Random Pace Inquiry*

In this mode of reasoning, the physician generates many “a posteriori” hypotheses by examining the patient’s information. But because of the unfocused nature of clinical inquiry, representation of the patient’s problem is lengthy and mostly irrelevant and because of unorganized nature of physician’s knowledge, these a posteriori hypotheses are numerous and usually wrong, and physician wanders among these hypotheses with aimless, frequent and fruitless bouts of trials and errors. 

At first, the physician takes a history of the patient (not focused on the problem), performs a physical examination. In this mode of inquiry, the patient’s problem is perceived as exhaustive and mostly irrelevant symbolically processed list of manifestations. The knowledge of such a person is highly irrelevant and poorly structured (dispersed knowledge). Dispersed knowledge and inadequate sensory of problem representation leads to frequent trials and errors around the diagnostic hypotheses. Diagnostic reasoning usually involves several iterations of random pace search for more clinical and Para-clinical cues. In this approach, the physician will order each possible test from the first contact with the patient (50). This shotgun test order is guided to some extent by recalled differential diagnoses (DDx). Unorganized knowledge about differential diagnoses, disease manifestations, and different diagnostic tests lead to the diagnostic hypotheses which the physician has made. Ultimately, after several bouts of trial and error, the physician may arrive at a diagnosis.

### *Exploratory Inquiry or main manifestation approach*

In this mode of reasoning, the physician seeks to generate a posteriori hypotheses by examining the patient’s information and looking for potential relations between different parts of that information. The physician usually focuses on the dominant manifestation (manifestation which is uncommon, clinically prominent, or with limited differential diagnoses (DDx) and thinks about differential diagnoses of the dominant manifestation.

The physician perceives the patient's problem as a list of manifestations, then he/she focuses on the main manifestation (M3 in figure 7) and considers differential diagnoses of the main manifestation (the initial and broadest list of hypotheses). The physician as the next step checks the differential diagnosis of the main manifestation (M3) for the presence of accessory manifestations (M1, M2, and M4 in figure 7) and generates a subset of DDx of the main manifestation which also covers accessory manifestations (secondary and limited list of hypotheses). In the final step, and after iterative rounds of patient exploration and revision (narrowing) of the list of hypotheses, the physician ultimately arrives at a satisfactory diagnosis. In this process, knowledge of differential diagnoses of manifestations and symptomatology of diseases helps the physician in generating and testing diagnostic hypotheses.

### *Focused Exploratory Inquiry or Shared Items Approach*

In this mode of reasoning, the physician seeks to generate a limited number of a posteriori hypotheses by examining the patient’s information. The physician frequently looks for shared items in DDX of different manifestations of the patient (intersection of sets of diseases in differential diagnoses of manifestations). Shared diseases in DDX of manifestations work as the initial list of hypotheses. The physician tries to narrow this list by applying their knowledge of diseases and iterative exploration of the patient.

The physician perceives the patient's problem as a short list of manifestations (focused manifestation) and considers differential diagnoses for these manifestations (M1, M2 in figure 8), then they uses their knowledge of manifestations stored in their knowledge structure to generate differential diagnoses. Then they find common items in the differential diagnosis of the manifestations (M1 ∩ M2), and generate intersection of DDx of manifestations (initial list of hypotheses). At the next step, the physician revises (narrows) the list of hypotheses through iterations of patient exploration until they arrive at a satisfactory diagnosis. This process is guided by the physician’s knowledge of diseases. 

### *Crucial Inquiry*

In this mode of reasoning, the physician decisively determines whether a particular hypothesis is superior to other competitive hypotheses. Usually, the patient’s problem is represented as a few numbers of competing a priori hypotheses. By looking for a few “discriminant-disease relevant” findings, the physician rapidly arrives at the final diagnosis. These inquiries are guided by relevant schemata. The schemata are composed of rules for further inquiries and subsequent steps according to the results (values) of each inquiry. Guided inquiries in each schema are set to assure largest information gain and the resultant findings have the largest classifying power.

The physician perceives the patient's problem as a short list of diseases and thinks about discriminant features of probable diseases (Diagnosis 1 (D1) vs. Diagnosis 2 (D2)), then they look for findings that can discriminate between hypotheses (e.g. D1 and D2), and this is done through the sequence that is led by the schema structure. At this stage, the physician uses conceptual knowledge of their schemas and ultimately arrives at a satisfactory diagnosis.

### *Confirmatory Inquiry*

In this mode of reasoning, the physician has a strong a priori hypothesis and is going to confirm his/her hypothesis by looking for a few findings that complete the problem representation into a satisfactory match with disease script.

In this type of inquiry, the physician's highly relevant clinical knowledge helps their decision. The patient’s problem is perceived as a single disease. Then he/she thinks about findings that can confirm the hypothesis (e.g. D1), and then he/she explores highly discriminant clinical findings. This inquiry is guided by deep conceptual knowledge of scripts until the physician arrives at a satisfactory diagnosis.

In each new clinical confrontation, the physician automatically uses the inquiry mode, which is more time efficient and requires the least mental effort. If this initial inquiry mode fails to arrive at an acceptable diagnosis, the physician shifts to less time efficient modes of inquiry. In different types of inquiries, if a physician cannot arrive at a satisfactory diagnosis, they use a lower level of inquiries in order to arrive at a diagnosis, and even if this inquiry does not lead to a satisfactory diagnosis, they use even a lower level of inquiry (Table 4).

**Table4:** The relationship between the components of cognitive zipper model and Drefyus stage of expertise

		Dreyfus stages of expertise					
		Novice	Advanced beginner	Competent	Proficient	Expert	Master
Main dimensions of diagnostic reasoning	Knowledge Structure	Reduced Knowledge	Dispersed Knowledge	Casual Knowledge	Elaborated casual Knowledge	Conceptual Knowledge	Deep conceptual Knowledge
	Problem representation	Inadequate sensory Representation	Symbolic Representation	Extended manifestation Representation	Focused manifestation Representation	Extended disease Representation	Focused disease Representation
	Inquiry	Dead End Inquiry	Random Pace Inquiry	Exploratory Inquiry	Focused Exploratory Inquiry	Crucial Inquiry	Confirmatory Inquiry

## Discussion

The previous claims of integration between different clinical reasoning models or development of a meta-model for clinical reasoning is restricted to few models from which the dual processing model is the most popular one. But these models suffer from a detailed explanation of clinicians’ cognitive processes during the act of clinical reasoning. Our model is consistent with psychological studies showing that the abstraction of the mental representation becomes increasingly more and more remote from the actual data ( 50 , 51 ) and, clinicians’ understanding of the problem and the problem representations is different ( 5 ).

It's necessary to note that forward and backward reasoning ( 52 , 53 ), and hypothetico-deductive and pattern recognition strategies are shown in the zipper model. In the first four modes of reasoning, which include dead end inquiry, random pace inquiry, an exploratory inquiry, and focused exploratory inquiry, the backward reasoning and hypothetico-deductive strategy are in progress, and in the two other modes of reasoning (crucial inquiry and confirmatory inquiry), the forward reasoning and pattern recognition strategy are in progress. Additionally, in the first four phases, a hypothetical strategy is used to make a diagnosis and in the final two stages, the pattern recognition is used. This is consistent with the findings of other studies that show that novices and experts have different patterns of data-driven and hypothesis-driven reasoning ( 27 ).

## Conclusion

We proposed three models in this study: the multi-layer knowledge structure model, problem representation model and cognitive zipper model of clinical reasoning. Multi-layer knowledge structure model clarified the differences between schema and script, and their formation. In addition, it determined the position of tacit and codified knowledge in knowledge structures. In the problem representation model, we defined types of the findings that form a problem representation. We used the multi-layer knowledge structure model and problem representation model to construct a comprehensive and novel model which describes clinical reasoning process. This model is cognitive zipper model. The proposed models have obvious advantages over other models proposed in the literature for explaining knowledge structures, problem representations and their interactions in the clinical reasoning process. In this model, we tried to clarify 1) the role of knowledge and its changes 2) explain about the formation of problem representation in physician’s mind in the process of clinical reasoning in both novice and expert clinicians. As the result of interactions between these two parts of clinical reasoning, the hypotheses are generated and these hypotheses are the core part of the clinical reasoning process. Constructing a model that captured the richness and complexity of clinical reasoning processes was very difficult, but we believe such a model would be very useful in teaching, learning, and assessment of clinical reasoning. This article described how this model was developed and how we illustrated it.
